# Energy-Efficient Wireless Multimedia Sensor Nodes for Plant Proximal Monitoring

**DOI:** 10.3390/s24248088

**Published:** 2024-12-18

**Authors:** Daniele Trinchero, Giovanni Paolo Colucci, Elena Filipescu, Ussama Syed Muhammad Zafar, Paola Battilani

**Affiliations:** 1 iXem Labs, Department of Electronics and Telecommunications (DET), Politecnico di Torino, 10129 Torino, Italy; giovanni.colucci@polito.it (G.P.C.); elena.filipescu@polito.it (E.F.); syed.zafar@polito.it (U.S.M.Z.); 2 Department of Sustainable Crop Production (DI.PRO.VE.S.), Università Cattolica del Sacro Cuore, 29122 Piacenza, Italy; paola.battilani@unicatt.it

**Keywords:** wireless multimedia sensor networks, camera wireless nodes, plant disease monitoring, LP-WAN, LoRa, LoRaWAN, GPRS, 2.5G mobile, IoT, smart agriculture, plant proximal monitoring, energy efficient multimedia nodes

## Abstract

The paper presents a double-radio wireless multimedia sensor node (WMSN) with a camera on board, designed for plant proximal monitoring. Camera sensor nodes represent an effective solution to monitor the crop at the leaf or fruit scale, with details that cannot be retrieved with the same precision through satellites or unnamed aerial vehicles (UAVs). From the technological point of view, WMSNs are characterized by very different requirements, compared to standard wireless sensor nodes; in particular, the network data rate results in higher energy consumption and incompatibility with the usage of battery-powered devices. Avoiding energy harvesters allows for device miniaturization and, consequently, application flexibility, even for small plants. To do this, the proposed node has been implemented with two radios, with different roles. A GPRS modem has been exclusively implemented for image transmission, while all other tasks, including node monitoring and camera control, are performed by a LoRaWAN class A end-node that connects every 10 min. Via the LoRaWAN downlink, it is possible to efficiently control the camera settings; the shooting times and periodicity, according to weather conditions; the eventual farming operations; the crop growth stages and the season. The node energy consumption has been verified in the laboratory and in the field, showing that it is possible to acquire one picture per day for more than eight months without any energy harvester, opening up further possible implementations for disease detection and production optimization.

## 1. Introduction

Crop phenology and crop status, with a focus on pest and disease occurrence, are among the key aspects that should be regularly monitored for the early detection of pests and diseases [1]. They are also part of the principles of integrated pest management [2]. As an example, the European Union regulation on Integrated Pest Management underlines regular inspection, early detection and diagnosis as mandatory for rational crop protection and a reduction in pesticide use [3].

Digital imaging allows to improve disease assessment compared to visual observation [1]; in fact, crop images have been widely used to track crop conditions and for the early detection of health problems to support decision making [4]. Image acquisition has been carried out primarily through satellites [5,6], for large-scale analyses, and unnamed aerial vehicles (UAVs) [7,8,9], for more localized inspections. The former allows to acquire images with a pixel definition down to 20 m with periodicity every 2–3 days, while the latter offers pixel definition down to 2–3 cm with periodicity only conditioned by costs. Unfortunately, both techniques do not offer the possibility to acquire high-definition images at the size of the leaf, fruit or flower.

Accurate disease outbreak detection is not easy with certain types of disease symptoms, such as small and evenly spaced spots, or symptoms with limited contrast, compared to the color of affected organs [10]. With traditional techniques, disease symptoms are commonly detected when they are well developed [11]. For this purpose, the scientific literature offers several machine learning and deep learning techniques to elaborate satellite and UAV images [12,13], but an effective alarm could only be retrieved with the application of high-definition cameras to a selection of properly identified testimonials. Moreover, in-crop camera-based monitoring could provide information about growth stages, plant morphology, foliage development, stress indicators and pest infestations [14,15,16].

Monitoring environmental parameters such as temperature and humidity is generally done on an hourly basis [17,18], while visual observation is commonly done with a 7-day or longer schedule [19,20] due to its cost [1,10]. Effectively, both plant and pathogen growth require one observation per day, as hourly and weekly periodicities are excessive and insufficient, respectively. For example, the season length of maize hybrids is defined by the number of days from crop emergence and generally varies from 100 to 135 days. Moreover, under optimal conditions, basil needs about 40 days from sowing to complete leaf development and 50 days for flowering [21]. Among fungi, *Peronospora belbahrii*, the causal agent of Peronospora in basil, has a latent period from infection to sporulation of 5–10 days, depending on the conduciveness of environmental conditions [22].

Consequently, one picture per day is sufficient for the majority of agronomical applications, but a differentiated scheduling strategy should be adopted for an efficient utilization of resources. The shooting time and the shooting parameters should be changed, according to weather conditions, phenological stages, sun positions, season variations, agronomic needs and farming activities that may compromise the image quality.

This paper introduces a novel device in the form of a Wireless Multimedia Sensor Node (WMSN) with a camera on board that wakes up every 10 min, transmits information about the node’s status and receives commands from remote. When a shooting command is received, the node activates the camera, takes the picture and transmits it to a central server. Energy consumption is optimized to require only a primary battery pack, minimizing dimensions, to allow deployment even in proximity to small plants. The objective is reached by doubling the radio interfaces. The result is a camera suitable for proximal monitoring applications: plant disease detection and phenology characterization.

## 2. State of the Art

WMSNs complement numerical sensor reading with images or videos, enhancing the overall monitoring and analysis processes [23].

Despite their advantages, WMSNs face several major challenges [24]:

- High bandwidth demand to transmit multimedia files;
- Need for efficient multimedia coding techniques to manage and compress data;
- Application-specific Quality-of-Service (QoS) requirements to ensure reliable and timely data transmission;
- Resource constraints, including limited battery life, energy harvesting, memory and processing power;
- In several countries, duty-cycle limitations in the sub-GHz radio-frequency (RF) band, as defined in ERC Recommendation 70-03 [25].

Among all, the main challenge is WMSNs’ energy consumption, especially when handling high-bandwidth data, such as images and videos [11]. The authors in [26] have emphasized the importance of developing energy-efficient protocols to extend the operational life of WMSNs.

During the past decades, various WMSN systems have been deployed, including the large-scale distributed system detailed in [27]. Efforts to reduce power consumption while managing discontinuous network connectivity have been explored. For instance, the authors in [28] have proposed a prioritizing buffer management algorithm to optimize energy use. In addition, a compression cost estimation scheme has been introduced in [29], which aims to balance power consumption and data quality, although it results in lower-resolution images.

Several authors have proposed the application of WMSNs to agriculture. The methodology described in [30] employs Internet of Things (IoT) technology for remote monitoring of crops and soil conditions. Images are collected once per week and an in-field network coordinator is required. In [18], data processing has been partially moved to the edge device, reducing network usage. The approach described in [31] uses LoRaWAN to transmit local image processing results to the cloud. The edge computing is done on a Raspberry Pi that requires a complex energy management.

Concerning the choice of the network standard, Low Power Networks like ZigBee and 6LowPAN [32,33] and Low Power Wide Area Networks (LP-WANs) like LoRa and Sigfox [34,35,36] do not offer data rates sufficient for multimedia data transmission. Some approaches have been proposed to parallelize LP-WAN data transmission, increasing the system complexity in terms of fail-over and synchronization [37].

Among mobile standards, GPRS is the most efficient technology, compared to 3G, LTE and 5G, which require more energy, unsuitable for the specific application. Moreover, GPRS has an important added value: When present, it is the mobile system with wider coverage in countryside and remote areas [23,38]. Unfortunately, mobile technologies are characterized by higher power consumption.

Short range, high data rate technologies such as Bluetooth Low Energy (BLE) and Wi-Fi [39,40,41] reduce flexibility, requiring the deployment of a dense network of receivers, limiting the applications of the device to greenhouse environments without any possibility for outdoor settings.

Table 1 summarizes the characteristics of the listed technologies, compared to the one proposed in this paper.

## 3. Materials and Methods

This paper introduces a battery-powered WMSN, able to take and transmit one picture per day, maintaining a frequent connection to the server for management purposes, and lasting one season on the same battery. We have chosen to avoid energy harvesters, to ensure miniaturization and a more compact system, suitable for plants of any size. To dimension the device, we have identified two distinct operational phases:

- Control phase (**C-phase**): device management from remote;
- Multimedia phase (**MM-phase**): picture shooting and transmission.

During the C-phase, the node sends an uplink containing monitoring parameters, including battery level information (2 bytes) and the last camera settings: autofocus (1 bit), autoexposure (1 bit), saturation (3 bits), brightness (4 bits) and resolution (4 bits), for a total of 4 bytes. The Received Signal Strength Indicator (RSSI) and Signal-to-Noise Ratio (SNR) are also available as metadata from the gateway. When the picture capture is requested, the server sends a downlink that contains the shooting command and the camera setup, for a total of 2 bytes. To comply with ERC 70-03 requirements, the uplink periodicity is set to 10 min.

Upon downlink reception, the MM-phase starts and the node performs a number of actions in sequence: It switches on the camera and the modem, it connects to the network, it sets up the camera, it takes the picture and it uploads it using a simple Transmission Control Protocol (TCP) socket.

**Figure 1:**
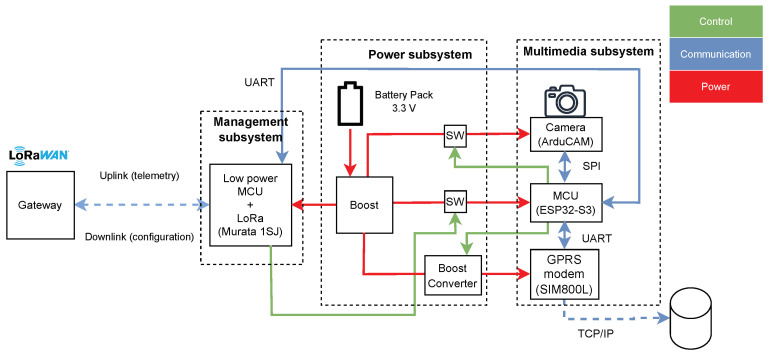
Block diagram of the device prototype, with the three subsystems: management subsystem on the left, power subsystem in the middle and multimedia subsystem on the right. Control connections are highlighted in green, communications channels in blue and power links in red.

To optimize the execution of the two phases, the device has been designed with three major components, as shown in Figure 1: management, multimedia and power subsystems.

The management subsystem has the responsibility for the C-phase as well as the activation/deactivation of the multimedia subsystem through the control of a switch in the power subsystem. It hosts an LP-WAN radio and a microcontroller (MCU); among the possible options, we have selected the LoRa technology and a LoRAWAN class A device. In particular, we have used a Murata 1SJ module, which embeds an STMicroelectronics MCU (STM32L0) and a LoRa Semtech radio (SX1262) [42]. The MCU is kept in deep sleep between two consecutive connections. When the MM-phase starts, the MCU activates the multimedia subsystem MCU, and then, it enters a sleep mode until the MM-phase process is completed; finally, it receives a feedback about the completion of the phase, switches off the multimedia subsystem MCU and enters a deep sleep state.

The multimedia subsystem has the responsibility of the MM-phase. It hosts the camera, the modem and the MCU, additional to the management subsystem one. The ESP32-S3 has been selected as the MCU, being well supported and having good computational power to process the picture. It is connected to the management subsystem MCU using serial communication (UART). As a radio, we have chosen GPRS technology, in particular, the SIM800L module, which can be controlled by AT commands sent over UART [43]. The camera has been implemented using the Arducam Mega SPI module, which has a compact size (33 mm × 33 mm) and is equipped with a standardized M12 lens screw-in mount that allows to use different lenses. The optical sensor acquires images with a resolution of 5 MP, automatically compressed in JPEG format.

The power subsystem hosts two AA primary alkaline batteries in series, followed by a boost to stabilize the battery voltage, two switches and one boost converter. One switch is controlled by the management subsystem MCU and used to activate/deactivate the multimedia subsystem MCU. The last one has the control of one additional switch that enables the camera and one boost converter that enables the GPRS modem, which needs a minimum of 4.1 V for correct operation.

The execution of the two phases by means of two separate subsystems is necessary. The GPRS standard is time demanding, especially its Packet Data Protocol (PDP) context activation, making it incompatible with 144 runs per day, unless an energy harvester is added. The LoRaWAN has a maximum payload incompatible with any image upload.

In the 2G and 2.5G mobile standards, the ability of the terminal (MT) to exchange data with the packet data network (PDN) requires the MT to establish a PDP with the Gateway GPRS Support Node (GGSN) [44,45]. This is done through the PDP context activation procedure, which is made up of several steps:

- Active PDP context request by the MT to the Serving GPRS Support Node (SGSN);
- Creation of the PDP context between the SGSN and the GGSN;
- Active Basic Service Set (BSS) packet flow context procedure management between the SGSN and the Base Station Controller (BSC);
- Update of the PDP context between the SGSN and the GGSN;
- Active PDP context acceptance by the SGSN to the MT.

The time requested for the execution of the listed commands depends on multiple factors and can take several seconds. In fact, the standard sets a timeout of 30 s before considering the procedure failed. For this reason, an average time equal to half timeout is assumed necessary to complete the PDP context activation after the MT switches on, with 5 more seconds necessary to exchange a data message [44].

This is confirmed in the literature, e.g., [46], where the minimum time necessary to complete a GPRS transmission after switching on the MT is reported to be equal to 12 s. We have also done several measurements, with different coverage and various traffic loads, measuring an average time of 16 s. Measurements have been performed by starting an internal timer and stopping it when the GPRS connection was completed successfully.

**Figure 2:**
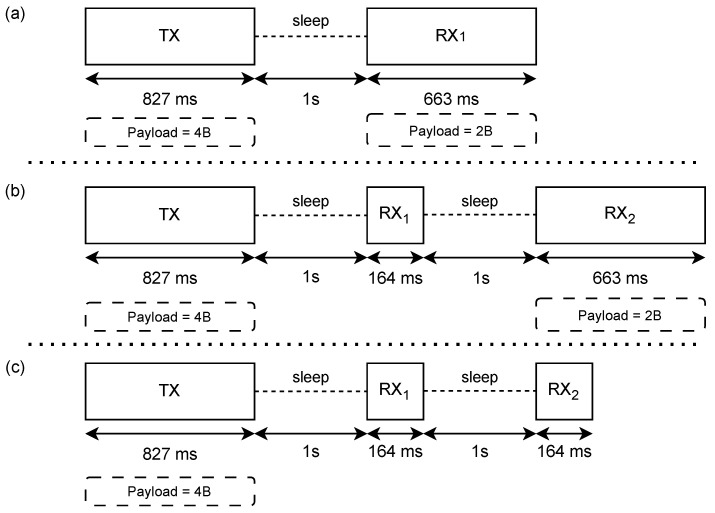
Time taken for an uplink/downlink sequence of a class A LoRaWAN end-node with spreading factor of 12, bandwidth of 125 kHz and coding rate of 4/5. (**a**) Downlink received in the first available window. (**b**) Downlink received in the second available window. (**c**) No downlink received.

On the contrary, a LoRaWAN class A end-node follows the timing scheme reported in Figure 2. After switching on, the node transmits a payload of 4 bytes occupying 827 ms, then, it waits for 1 s before opening a first receiving window that lasts 164 ms (if no downlink is detected) or a time necessary to receive the 2 bytes. In case the downlink is not detected, after 1 more second, a second receiving window is opened, lasting a minimum of 164 ms (in case no downlink is detected) or a time necessary to receive the 2 bytes.

The time required to receive a minimal 2 byte payload command differs significantly between the GPRS and LoRaWAN standards, with the former being more than four times longer. This suggests the need to double the radios, introducing two separate subsystems.

## 4. Results

Once constructed, the device has been initially characterized in laboratory, where we have measured its energy consumption. In particular, we have experimentally evaluated the energy requested to complete the C-phase 144 times per day, comparing the proposed solution to a standard GPRS solution. Then, we have estimated the energy to execute 1 MM-phase per day. After, we have estimated the daily energy need, by summing the current absorbed by the management subsystem MCU in deep sleep mode. This has allowed us to correctly dimension the battery pack. Finally, we have put the device into field operation for its validation.

### 4.1. Energy Consumption

**Figure 3:**
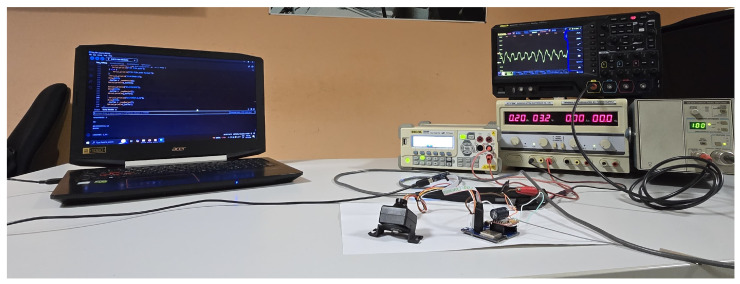
Current probe amplifier and digital oscilloscope used to measure current consumption. A 3.3 V voltage has been provided by a regulated DC power supply.

The current consumption has been measured using a Tektronix AM 503 B current probe amplifier [47] and a RIGOL MSO5104 digital oscilloscope [48]. The measurement setup is shown in Figure 3.

During the C-phase execution, the node has been cautiously configured to transmit the maximum power: 14 dBm. The absorbed current has been measured during the transmission of the uplink and the reception of the downlink, obtaining 90.2 mA and 10.1 mA, respectively. Considering the timing scheme of Figure 2, the charge required for the uplink is 20.72 μA h, the charge required for the short receiving window is 0.46 μA h and the charge required for the long receiving window is 1.86 μA h. Since the node is expected to connect every 10 min, receiving one downlink per day, we have made a precautionary assumption: It daily operates 143 times in mode (c) and 1 time in mode (b), corresponding to 3.12 mA h per day. The results are summarized in Table 2.

The same instrumental setup has been used to measure the current necessary to power a GPRS node performing the same actions. In this case, with a total duration of 20 s, an average current equal to 95 mA is necessary, leading to 0.53 mA h per connection, consuming a total charge of 76 mA h per day.

Table 3 shows, for each of the five consecutive steps of the MM-phase, the absorbed current, the duration and the energy consumption. Initially, the MCU is powered on, then it switches on the GPRS modem and the camera; while the former establishes the connection to the network, the latter is set with the parameters received by the management subsystem MCU via UART. The next interval is the picture shooting, which draws the highest current, although it lasts only 0.066 s. After, the picture is sent through the GPRS modem. We have measured the time requested to transmit a 200 kB image, under different coverage conditions, forcing varying traffic congestion, obtaining an average value of 25 s. The last activity is the transmission of the feedback to the management subsystem, via UART. During the whole MM-phase, the management subsystem MCU remains in sleep mode, absorbing 5 mA, corresponding to additional 0.05 mA h. As a result, the total amount of charge needed to complete the MM-phase is equal to almost 4 mA h.

Finally, we have measured the current absorbed by Murata 1SJ when in deep sleep mode and obtained 50 μA, which corresponds to 1.2 mA h per day.

As it is shown in Table 4, summing the charge needed for 144 C-phase executions, 1 MM-phase execution and Murata 1SJ deep sleep, the proposed device needs 8.31 mA h per day. Using a battery pack with a series of two AA alkaline batteries with a capacity of 2500 mA h, 300 days are, theoretically, allowed. If a GPRS solution was used, almost 91.59 mA h per day would be necessary, and the same battery pack would have been sufficient for no more than 27 days.

### 4.2. Field Validation

**Figure 4:**
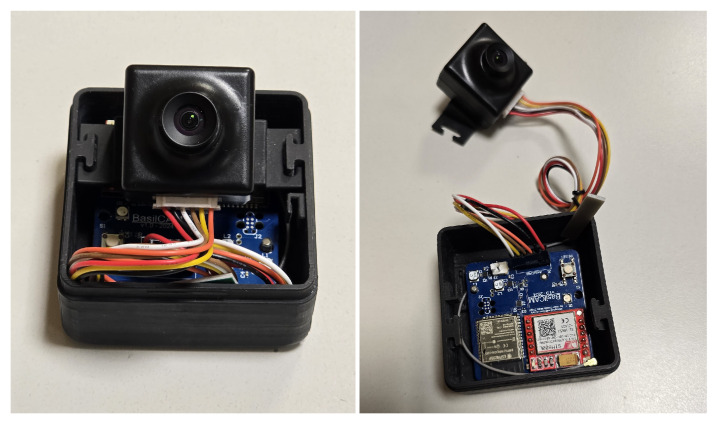
The device prototype inside the 3D-printed case: The battery pack is placed on the bottom layer; the case lid has been removed and the camera has been extracted for a better view of the details.

For its in-field validation, a device prototype has been manufactured, as it is shown in Figure 4. The case has been 3D printed using a Formlab—Form 3 printer with black resin. Inside, the battery pack is deployed on the bottom, the Printed Circuit Board (PCB) in the middle and the camera on top. The case lid is made of acrylic glass, to allow transparency. The prototype has been tested with a set of different lenses for the camera, with a Horizontal Field of View (HFoV) of 10, 26, 33, 67, 73, 100, 118, 141, 180 and 220 degrees. Smaller values of FoV have been used for a detailed picture, at the expense of the photographed area, while higher values of FoV have been used to capture larger images with a decreased level of detail.

**Figure 5:**
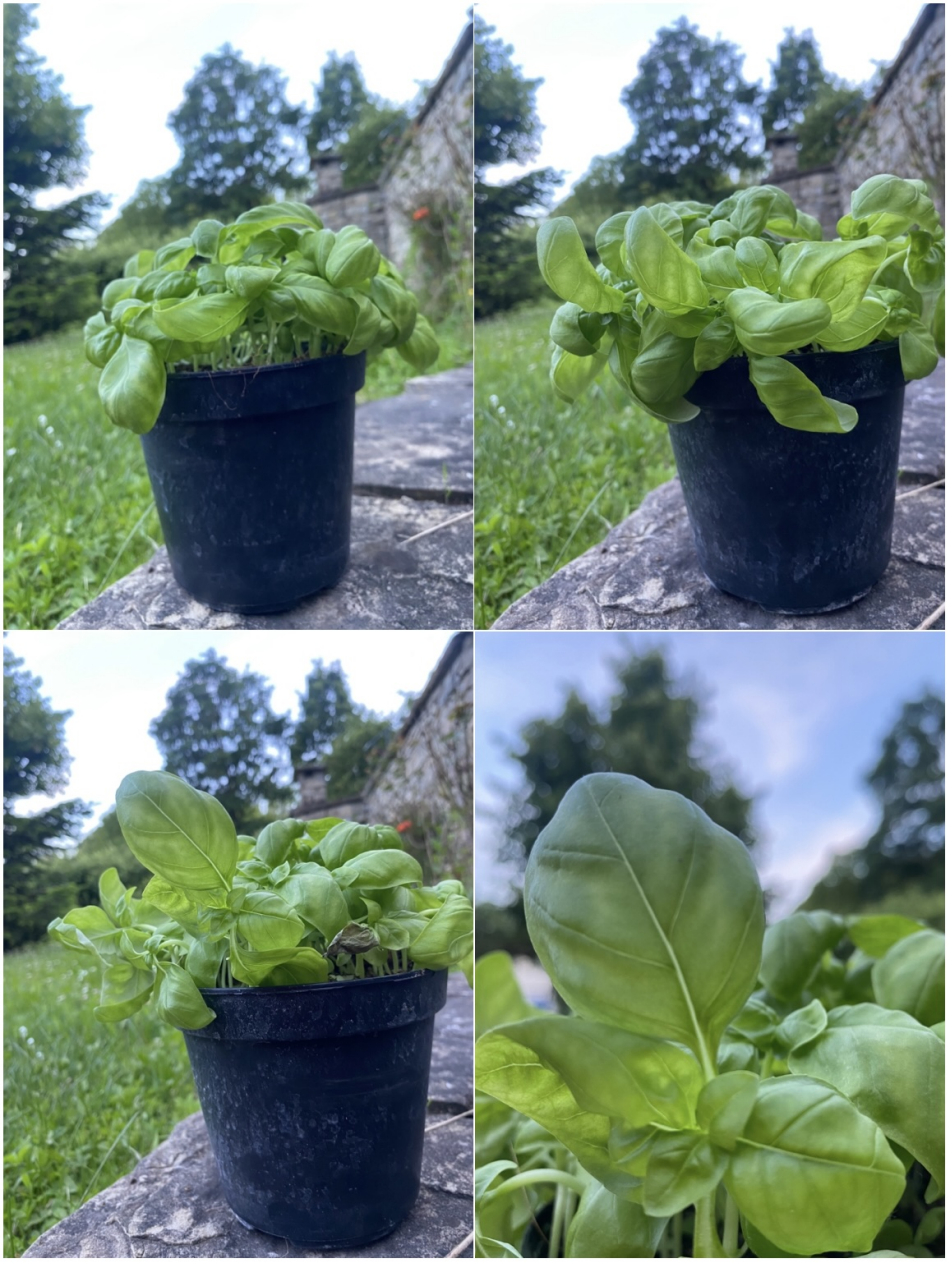
Potted basil plants pictures taken with the prototype device. **Top left**: early growth stage (lens 73° HFoV); **top right**: intermediate growth stage (lens 73° HFoV); **bottom left**: late growth stage (lens 73° HFoV); **bottom right**: picture taken with different lens selection (lens 33° HFoV).

Then, it has been then validated using a basil pot as shooting target, deployed at the entrance of a farm located in Verrua Savoia (Italy). During the validation period, the area has been exposed to frequent rain showers, making it possible to combine the picture acquisition with the automatic adaptation of the shooting schedule. Figure 5 shows results taken with the first implemented prototype. The first three images (top left, top right and bottom left) are taken in correspondence of different growth stages: early phase, 28 April, early morning; intermediate phase, 5 May, midafternoon; and late phase, 15 May, early evening. For the shootings, the lens with 73° HFoV was used. The last image (bottom right) is taken with lens 33° HFoV.

## 5. Conclusions

The key innovation of the proposed design is represented by the insertion of two subsystems, each containing an MCU and a radio, with the former dedicated to the multimedia processing/transmission and the latter to the node control from remote. It strategically exploits both GPRS and LoRaWAN technologies to balance the higher data rate requirements for image transmission with the energy efficiency demands for an almost continuous connection. The device can be adapted to varying weather conditions, agronomic needs and farming activities. This flexibility ensures image capture at optimal times, improving data relevance and quality.

The modular approach facilitates the customization for different use cases and simplifies the integration of additional sensors, e.g., an air temperature and humidity sensor, or functionalities. This hardware can be integrated through digital or analog breakout available on the board. This adaptability is vital for addressing the various needs of the prototyping phase.

In terms of security, LoRaWAN implements, by design, encryption (AES-CTR-128), data integrity (AES-CMAC-128) and authentication. The 128 bit key size is sufficient for LP-WAN applications. Instead, the lack of modern security standards in GPRS has been balanced by enforcing end-to-end security in the application layer, exploiting FTPS that leverages TLS to guarantee security. Defense against physical tampering has not been included, but may be adopted when deploying the device on a larger scale in less controlled environments.

Future works will focus on field testing and validation of the system under different agronomic conditions. Tests of LoRaWAN performance will be conducted on a large scale by deploying gateways, collecting metrics and evaluating the overall reliability. Furthermore, to extend the device lifetime, alternative battery solutions, such as thionyl chloride ones, could be investigated and adopted after security tests in the laboratory and in the field. The computational capacity of the ESP32-S3 could be exploited with one of the many machine learning models proposed in the literature for plant monitoring. On-board processing could be used to choose whether to send a picture or not, activating GPRS transmission only when strictly necessary, improving battery duration. By running image processing algorithms locally, it would be possible to overcome the rare limitations occurring in the absence of GPRS coverage, assigning to the management subsystem the role to activate agronomic alarms.

## Figures and Tables

**Table 1:** Summary of the characteristics of the existing systems, compared to the proposed device.

Technology	Key Advantages	Limitations
ZigBee/6LowPAN	Low power	Not suitable for multimedia data transmission
LoRa/Sigfox	Wide coverage and ultra-low power	Low data rates, not suitable for multimedia data transmission
GPRS	Extensive coverage in rural areas	High energy consumption
BLE/Wi-Fi	High data rate	Requires a dense receiver network, unsuitable for outdoor deployment
Proposed Device	Low power, high data rate	Requires GPRS coverage

**Table 2:** Energy necessary for the execution of the C-phase: A comparison between the proposed device and a full GPRS implementation.

	Average Current (mA)	Time (s)	Total Energy Consumption (mA h)
Uplink	90.2	0.827	0.0207
Short Receive Window	10.1	0.164	0.00046
Long Receive Window	10.1	0.663	0.00186
GPRS	95.0	20.000	0.53

**Table 3:** Absorbed current, time duration and energy necessary for the execution of the five consecutive steps of the MM-phase.

	Average Current (mA)	Average Time (s)	Total Energy Consumption (mA h)
MCU activation	87.3	0.003	∼0
Modem activation and network connection	98.0	15.000	0.4
Picture shooting	571.8	0.066	0.0096
Picture transmission	503.9	25.000	3.125
Feedback transmission	87.3	0.010	∼0

**Table 4:** Daily charge necessary to complete the node operations: C-phase, MM-phase and deep sleep. Comparison between the proposed device and a full GPRS solution.

Device	C-Phase (mA h)	MM-Phase (mA h)	Deep Sleep (mA h)	Total (mA h)	Days
Proposed device	3.12	4	1.2	8.31	300
GPRS	76.08	4	11.6	91.59	27

## Data Availability

The raw data supporting the conclusions of this article will be made available by the authors on request.

## Abbreviations

The following abbreviations are used in this manuscript:

UAV	Unnamed Aerial Vehicle
WMSN	Wireless Multimedia Sensor Network
QoS	Quality of Service
RF	Radio Frequency
ERC	European Radiocommunication Committee
IoT	Internet of Things
LoRaWAN	Long-Range Wide-Area Network
LP-WAN	Low-Power Wide-Area Network
LoRa	Long Range
GPRS	General Packet Radio Service
LTE	Long-Term Evolution
BLE	Bluetooth Low Energy
C-phase	Control phase
MM-phase	Multimedia phase
RSSI	Received Signal Strength Indicator
SNR	Signal-to-Noise Ratio
TCP	Transmission Control Protocol
MCU	MicroController Unit
UART	Universal Asynchronous Receiver-Transmitter
AT	ATtention
SPI	Serial Peripheral Interface
PDP	Packet Data Protocol
MT	Mobile Terminal
PDN	Packet Data Network
GGSN	Gateway GPRS Support Node
SGSN	Serving GPRS Support Node
BSS	Basic Service Set
BSC	Base Station Controller
FTPS	File Transfer Protocol Secure
TLS	Transport Layer Security
PCB	Printed Circuit Board
HFoV	Horizontal Field of View
FoV	Field of View

## References

[1] Mutka A.M., Bart R.S. (2015). Image-based phenotyping of plant disease symptoms. Front. Plant Sci..

[2] Barzman M., Bàrberi P., Birch A.N.E., Boonekamp P., Dachbrodt-Saaydeh S., Graf B., Hommel B., Jensen J.E., Kiss J., Kudsk P. (2015). Eight principles of integrated pest management. Agron. Sustain. Dev..

[3] European Union (2009). Directive 2009/136/EC of the European Parliament and of the Council of 25 November 2009 Amending Directive 2002/22/EC on Universal Service and Users’ Rights Relating to Electronic Communications Networks and Services, Directive 2002/58/EC Concerning the Processing of Personal Data and the Protection of Privacy in the Electronic Communications Sector and Regulation (EC) No 2006/2004 on Cooperation Between National Authorities Responsible for the Enforcement of Consumer Protection Laws. https://eur-lex.europa.eu/LexUriServ/LexUriServ.do?uri=OJ:L:2009:309:0071:0086:en:PDF.

[4] Kamilaris A., Prenafeta-Boldú F.X. (2018). Deep learning in agriculture: A survey. Comput. Electron. Agric..

[5] Karthikeyan L., Chawla I., Mishra A.K. (2020). A review of remote sensing applications in agriculture for food security: Crop growth and yield, irrigation, and crop losses. J. Hydrol..

[6] Sishodia R.P., Ray R.L., Singh S.K. (2020). Applications of Remote Sensing in Precision Agriculture: A Review. Remote Sens..

[7] Segarra J., Buchaillot M.L., Araus J.L., Kefauver S.C. (2020). Remote Sensing for Precision Agriculture: Sentinel-2 Improved Features and Applications. Agronomy.

[8] Tsouros D.C., Bibi S., Sarigiannidis P.G. (2019). A Review on UAV-Based Applications for Precision Agriculture. Information.

[9] Velusamy P., Rajendran S., Mahendran R.K., Naseer S., Shafiq M., Choi J.G. (2022). Unmanned Aerial Vehicles (UAV) in Precision Agriculture: Applications and Challenges. Energies.

[10] Bock C., Poole G., Parker P., Gottwald T. (2010). Plant disease severity estimated visually, by digital photography and image analysis, and by hyperspectral imaging. Crit. Rev. Plant Sci..

[11] Zaineldin H., Elhosseini M., Ali H. (2014). Image compression algorithms in wireless multimedia sensor networks: A survey. Ain Shams Eng. J..

[12] Barbedo J.G.A. (2016). A review on the main challenges in automatic plant disease identification based on visible range images. Biosyst. Eng..

[13] Abade A., Ferreira P.A., de Barros Vidal F. (2021). Plant diseases recognition on images using convolutional neural networks: A systematic review. Comput. Electron. Agric..

[14] Correa E.S., Calderon F.C., Colorado J.D. (2024). A Novel Multi-camera Fusion Approach at Plant Scale: From 2D to 3D. SN Comput. Sci..

[15] Wang Y., Rajkumar Dhamodharan U.S., Sarwar N., Almalki F.A., Naith Q.H., R S., D M. (2024). A Hybrid Approach for Rice Crop Disease Detection in Agricultural IoT System. Discov. Sustain..

[16] Kondaparthi A.K., Lee W.S., Peres N.A. (2024). Utilizing High-Resolution Imaging and Artificial Intelligence for Accurate Leaf Wetness Detection for the Strawberry Advisory System (SAS). Sensors.

[17] Surige Y., Perera W., Gunarathna P., Ariyarathna K., Gamage N., Nawinna D. IoT-Based Monitoring System for Oyster Mushroom Farming. Proceedings of the 2021 3rd International Conference on Advancements in Computing (ICAC).

[18] Nguyen H.H., Shin D.Y., Jung W.S., Kim T.Y., Lee D.H. (2024). An Integrated IoT Sensor-Camera System toward Leveraging Edge Computing for Smart Greenhouse Mushroom Cultivation. Agriculture.

[19] Lovell D., Powers S., Welham S., Parker S. (2004). A perspective on the measurement of time in plant disease epidemiology. Plant Pathol..

[20] Gordy J.W., Seiter N.J., Kerns D.L., Reay-Jones F.P.F., Bowling R.D., Way M.O., Brewer M.J. (2021). Field Assessment of Aphid Doubling Time and Yield of Sorghum Susceptible and Partially Resistant to Sugarcane Aphid (Hemiptera: Aphididae). J. Econ. Entomol..

[21] Aldarkazali M., Rihan H.Z., Carne D., Fuller M.P. (2019). The Growth and Development of Sweet Basil (*Ocimum basilicum*) and Bush Basil (*Ocimum minimum*) Grown under Three Light Regimes in a Controlled Environment. Agronomy.

[22] Cohen Y., Ben Naim Y., Falach L., Rubin A.E. (2017). Epidemiology of basil downy mildew. Phytopathology.

[23] Prathibha S.R., Hongal A., Jyothi M.P. IOT Based Monitoring System in Smart Agriculture. Proceedings of the 2017 International Conference on Recent Advances in Electronics and Communication Technology (ICRAECT).

[24] Al Nuaimi M., Sallabi F., Shuaib K. A survey of Wireless Multimedia Sensor Networks Challenges and Solutions. Proceedings of the 2011 International Conference on Innovations in Information Technology.

[25] (2022). Relating to the Use of Short Range Devices (SRD).

[26] Akyildiz I.F., Melodia T., Chowdhury K.R. (2007). A survey on wireless multimedia sensor networks. Comput. Netw..

[27] Campbell J., Gibbons P.B., Nath S., Pillai P., Seshan S., Sukthankar R. IrisNet: An Internet-Scale Architecture for Multimedia Sensors. Proceedings of the 13th Annual ACM International Conference on Multimedia.

[28] Feng W.C., Kaiser E., Feng W.C., Baillif M.L. (2005). Panoptes: Scalable low-power video sensor networking technologies. ACM Trans. Multimed. Comput. Commun. Appl..

[29] Zhang Q.Y., Huang H.P., Sha C. An Energy Efficient Image Transmission Scheme for Wireless Multimedia Sensor Networks. Proceedings of the 2012 Fourth International Conference on Computational and Information Sciences.

[30] Savin I.Y., Blokhin Y.I., Chinilin A.V. (2024). Methodology of Real-time Monitoring of the Crop Status Based on Internet of Things Technologies. Russ. Agric. Sci..

[31] Chamara N., Bai G.F., Ge Y. (2023). AICropCAM: Deploying classification, segmentation, detection, and counting deep-learning models for crop monitoring on the edge. Comput. Electron. Agric..

[32] Baronti P., Pillai P., Chook V.W., Chessa S., Gotta A., Hu Y.F. (2007). Wireless sensor networks: A survey on the state of the art and the 802.15.4 and ZigBee standards. Comput. Commun..

[33] Garcia-Sanchez A.J., Garcia-Sanchez F., Garcia-Haro J. (2011). Wireless sensor network deployment for integrating video-surveillance and data-monitoring in precision agriculture over distributed crops. Comput. Electron. Agric..

[34] Liya M., Aswathy M. LoRa Technology for Internet of Things(IoT):A Brief Survey. Proceedings of the 2020 Fourth International Conference on I-SMAC (IoT in Social, Mobile, Analytics and Cloud) (I-SMAC).

[35] Almuhaya M.A.M., Jabbar W.A., Sulaiman N., Abdulmalek S. (2022). A Survey on LoRaWAN Technology: Recent Trends, Opportunities, Simulation Tools and Future Directions. Electronics.

[36] Chochul M., Ševčík P. A Survey of Low Power Wide Area Network Technologies. Proceedings of the 2020 18th International Conference on Emerging eLearning Technologies and Applications (ICETA).

[37] Wei C.C., Chen S.T., Su P.Y. Image Transmission Using LoRa Technology with Various Spreading Factors. Proceedings of the 2019 2nd World Symposium on Communication Engineering (WSCE).

[38] Zhang Z., Wu P., Han W., Yu X. (2017). Remote monitoring system for agricultural information based on wireless sensor network. J. Chin. Inst. Eng..

[39] Lloret J., Garcia M., Bri D., Sendra S. (2009). A Wireless Sensor Network Deployment for Rural and Forest Fire Detection and Verification. Sensors.

[40] Mukherjee D., Das A., Ghosh N., Nanda S. Real Time Agricultural Monitoring with Deep Learning Using Wireless Sensor Framework. Proceedings of the 2023 International Conference on Electrical, Electronics, Communication and Computers (ELEXCOM).

[41] Chang K.C., Liu P.K., Kuo Z.W., Liao S.H. Design of Persimmon Growing Stage Monitoring System Using Image Recognition Technique. Proceedings of the 2016 IEEE International Conference on Consumer Electronics-Taiwan (ICCE-TW).

[42] Murata Type 1SJ LPWA Modules. https://www.murata.com/en-eu/products/connectivitymodule/lpwa/overview/lineup/type-1sj.

[43] Adafruit Industries LLC (2013). SIM800 Series AT Command Manual. https://www.digikey.jp/htmldatasheets/production/1833952/0/0/1/sim800-series-at-command-manual.html#pfdf.

[44] 3rd Generation Partnership Project (3GPP) 3GPP TS 24.008 V13.7.0 (2016-10): Mobile Radio Interface Layer 3 Specification; Core Network Protocols; Stage 3. Technical Specification 3GPP TS 24.008, 3rd Generation Partnership Project (3GPP), 2016, ETSI, Sophia Antipolis Cedex, France.

[45] Telit Communications 2G/3G/4G Registration Process. Application Note Revision 3, Telit Communications, London, UK, 2021. https://sixfab.com/wp-content/uploads/2022/01/Telit_2G_3G_4G_Registration_Process_Application_Note_r3.pdf.

[46] Tunccekic Y., Dincer K. (2007). Mobile Mapping Applications over J2ME Enabled Phones. IJCSNS Int. J. Comput. Sci. Netw. Secur..

[47] Tektronix (1994). AM 503B & AM 5030 AC/DC Current Probe Amplifiers.

[48] RIGOL Technologies EU GmbH (2020). RIGOL MSO5104 User Guide. https://rigolshop.eu/product-oscilloscope-mso5000-mso5104.html#amcustomtabs_tabs_10.

